# Q Fever Update, Maritime Canada

**DOI:** 10.3201/eid1401.071256

**Published:** 2008-01

**Authors:** Thomas J. Marrie, Nancy Campbell, Shelly A. McNeil, Duncan Webster, Todd F. Hatchette

**Affiliations:** * University of Alberta, Edmonton, Alberta, Canada; † QEII Health Sciences Centre, Halifax, Nova Scotia, Canada

**Keywords:** Q fever, Coxiella burnetii, pneumonia, Nova Scotia, New Brunswick, Prince Edward Island, Canada, dispatch

## Abstract

Since the 1990s, reports of Q fever in Nova Scotia, Canada, have declined. Passive surveillance for Q fever in Nova Scotia and its neighboring provinces in eastern Canada indicates that the clinical manifestation of Q fever in the Maritime provinces is pneumonia and that incidence of the disease may fluctuate.

The first cases of Q fever in Nova Scotia were recognized in 1979 during a study of atypical pneumonia (*1*). This observation led to a series of studies that showed that Q fever was common in Nova Scotia (50–60 cases per year in a population of ≈950,000) and that the epidemiology was unique; exposure to infected parturient cats or newborn kittens was the major risk factor for infection (*2*). At about the same time, cat-related outbreaks were noted in neighboring Prince Edward Island and New Brunswick (*2*). In the early 1990s, cases began to decline; but, to our knowledge, since 1999 Q fever in this area has not been systematically studied. We undertook the current study to determine whether Q fever was still occurring in Maritime Canada (the 3 provinces of New Brunswick, Nova Scotia, and Prince Edward Island in eastern Canada) and whether the decline in cases was real or an artifact of decreased surveillance.

## The Study

The study began in December 2004 and terminated on February 17, 2007. A notice sent to all physicians in Maritime Canada described the study and asked the physicians to submit serum samples from patients with suspected Q fever (febrile illness or pneumonia after exposure to parturient cats or other animals; outbreaks of pneumonia in a family). All samples were sent to the Nova Scotia Public Health Laboratory for testing for antibodies to *Coxiella burnetii*. Physicians of patients with a positive test result were contacted, and they in turn contacted their patients to ask if they would participate in the study. This study was approved by the University of Alberta Research Ethics Review Board and the Capital Health Research Ethics Board.

During the study period, serum samples from 210 patients suspected of having *C. burnetii* infection were tested by using commercially available immunoglobulin (Ig) M and IgG ELISAs (PanBio, Brisbane, Queensland, Australia). Patients with positive IgM ELISA results were asked to participate in the study. Convalescent-phase samples were collected from those who agreed; further testing for IgG antibodies to phase I and phase II *C. burnetii* antigen was conducted by using an indirect immunofluorescence test as previously described (*3*).

Of the 210 patients, 35 had antibodies to *C. burnetii* and 13 met the criteria for acute Q fever (positive IgM ELISA result and a >4-fold rise in antibody to phase II antigen between the acute- and convalescent-phase samples). Phase I titers >512, suggestive of chronic Q fever, were found for 3 patients. The other 19 patients had serologic profiles suggestive of previous exposure to *C. burnetii*. Of the 13 patients who fit the case definition of acute infection, 11 agreed to participate in the study, 1 declined, and 1 moved to another country.

Of the 11 participating patients, 7 were from Nova Scotia, 2 were from New Brunswick, and 2 were from Prince Edward Island; 6 were male; and mean age was 54.6 years (Table). One case occurred in December 2004; 6 in 2005; 4 in 2006, and no cases in the first 6 weeks of 2007. Cases occurred in every month except August, September, October, and February.

**Table:** Selected features of 11 patients with acute Q fever, Maritime Canada

Patient	Date of onset	Sex	Age, y	Hospitalized	Pneumonia	Risk factors
1	2005 Jan 10	F	42	No	Yes	Farmer*
2	2004 Nov 1	F	50	Yes	Yes	Farm visit
3	2005 July 18	F	67	Yes	ND†	Farm visit
4	2005 Apr 17	M	56	Yes	No	Farm visit, deer hunting
5	2006 May 24	M	44	Yes	Yes	Newborn calves
6	2006 June 30	F	51	No	Yes	None
7	2005 Dec 31	M	59	No	Yes	Newborn lambs
8	2006 May 22	M	57	No	Yes	Newborn lambs
9	2005 Mar 1	F	50	No	ND†	Parturient cat and her kittens
10	2005 Mar 1	M	69	No	ND†	Parturient cat and her kittens
11	2006 Jan 1	M	56	Yes‡	Yes	Newborn poodles, livestock auction

* Within 2 weeks of onset of symptoms, this patient had occupied a small space (automatic bank teller area) with 2 farmers who smelled of manure. †ND, not determined because chest radiographs were not taken. ‡Required intubation.

All patients except patient 6 had risk factors for Q fever (Table). One patient (patient 8) was a sheep farmer who had recently had ≈60 lambs born on his farm, several of which were stillborn in the 2 weeks before the farmer became ill; 7 patients had >1 cat as a pet; and only 2 (patients 5 and 8) had no pets.

In terms of clinical signs, all 11 patients had sweats, fever, and myalgia; 9 had chills; 8 had a cough; 7 were short of breath; 5 each had nausea, diarrhea, sputum production, and confusion; 4 had chest pain, which was pleuritic for 2; and 2 had abdominal pain and vomiting. Of the 7 patients for whom chest radiographs were taken, 6 had acute opacities compatible with pneumonia. Patient 11 had diffuse bilateral pneumonia, which required him to be admitted to an intensive care unit to receive ventilatory support (Figure).

**Figure:**
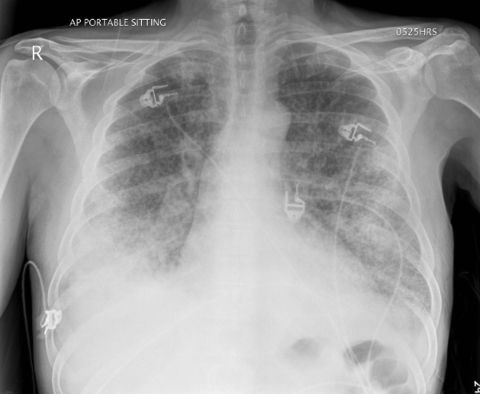
Chest radiograph of patient 11 at time of admission to hospital, before intubation, demonstrating extensive bilateral airspace disease.

All 11 patients recovered. Only 4 received initial empiric therapy that would be considered effective against *C. burnetii,* e.g., doxycycline (n = 2), ciprofloxacin (n = 1), or levofloxacin (n = 1). Four other patients received azithromycin, which may have been effective but has suboptimal in vitro activity against *C. burnetii* (*4*).

## Conclusions

Acute Q fever is still present in Maritime Canada; however, the number of cases has diminished considerably from the 1980s and early 1990s. Since 2004, only 4–5 cases have been reported each year. The passive design of our study may have underestimated the number of cases. However, in the 1980s, a number of Q fever outbreaks involved entire families. A typical scenario was exposure to the parturient family cat and her newborn kittens, after which everyone in the family became ill (*2*). Some outbreaks involved poker players (*5*) or most of the employees of a factory (*6*). For our study, we carefully asked whether family members were ill; only 2 patients mentioned such illness, and for each, it was a spouse.

Pneumonia seems to still be the dominant form of acute Q fever in Maritime Canada. Of the 7 patients for whom chest radiographs were taken, 6 had pneumonia. Major differences in the manifestations of Q fever occur in different regions. In Maritime Canada and in the Basque region of Spain, the predominant manifestation is pneumonia (*7*,*8*); in Newfoundland and Australia, fever with no apparent localization of infection (*9*,*10*); in the Canary Islands, fever and hepatitis; and in southern France, hepatitis and pneumonia, although hepatitis is more frequent than pneumonia (*11*,*12*). The factors responsible for these disparate manifestations are not known. When isolates of *C. burnetii* from different geographic areas were typed by using multisequence typing, all 7 isolates from Nova Scotia were identical and shared this type with 2 isolates from France and 1 from the United States (*13*).

The reservoirs for human infection with *C. burnetii* in Nova Scotia have likely spread from cats and dogs (*14*) to the more traditional reservoirs of sheep and cattle (*12*). Patient 8, a sheep farmer, had pneumonia that appeared on radiographs as a rounded opacity in the right middle lobe. Rounded opacities are very common in cat-associated cases of Q fever but may not be specific for cat-associated infection (*15*).

Our findings indicate that after *C. burnetii* is established in an area, it is likely to persist, although the incidence may fluctuate. Clinical manifestations, which in our study were limited to pneumonia, remain stable.
